# Supplementary material for: Mechanobiochemical finite element model to analyze impact-loading-induced cell damage, subsequent proteoglycan loss, and anti-oxidative treatment effects in articular cartilage
Source: Biomech Model Mechanobiol. 2025 May 10;24(4):1191–206. doi: 10.1007/s10237-025-01961-8 (PMC12246027; doi:10.1007/s10237-025-01961-8)
Supplement: Supplementary file 1 — Supplementary file1 (DOCX 2388 KB) [file 10237_2025_1961_MOESM1_ESM.docx]

**Electronic Supplementary material**

For “*Mechanobiochemical finite element model to analyze impact-loading-induced cell damage, subsequent proteoglycan loss, and anti-oxidative treatment effects in articular cartilage*) by

Joonas P. Kosonen^1^*, Atte S.A. Eskelinen^1^, Gustavo A. Orozco^1^, Donald D. Anderson^2^, Mitchell C. Coleman^2^, Jessica E. Goetz^2^, Alan J. Grodzinsky^3^, Petri Tanska^1^ and Rami K. Korhonen^1^

^1^*Department of Technical Physics, University of Eastern Finland, Kuopio, Finland*

*^2^Departments of Orthopedics & Rehabilitation and Biomedical Engineering, University of Iowa, Iowa City, IA, United States of America*

*^3^Departments of Biological Engineering, Electrical Engineering and Computer Science, and Mechanical Engineering, Massachusetts Institute of Technology, Cambridge, United States of America*

**Corresponding author:**

*Joonas P. Kosonen

Department of Technical Physics

University of Eastern Finland

Yliopistonranta 8

POB 1627, Kuopio FI-70211, Finland

Tel: +358 50 3043474

[joonas.kosonen@uef.fi](mailto:attees@uef.fi)

**S1 Structural, compositional, and material inputs for the biomechanical simulations of the modeling framework**

The biomechanical response of the cartilage to the drop-tower impact was simulated in Abaqus, where cartilage was modeled with fibril-reinforced poroviscoelastic material model with Donnan osmotic swelling as described previously (Wilson et al. 2004, 2005b). The structural, compositional, and material patameters utilized in the model (Eq. (1)-(10)) are presented in table S1.

**Table S1.** Structural, compositional and material parameters to simulate impact loading of mature bovine cartilage explants.

| **Biomechanical model to simulate impact** | | | |
| --- | --- | --- | --- |
| **Structural parameters** | Symbol | Value | Reference |
| Sample thickness | *H* [mm] | 1.0 | (Martin et al. 2009) |
| Surface layer thickness | *d*_sup_ [mm] | 0.11H | (Julkunen et al. 2007) |
| Middle layer thickness | *d*_mid_ [mm] | 0.17H | (Julkunen et al. 2007) |
| Deep layer thickness | *d*_deep_ [mm] | 0.72H | (Julkunen et al. 2007) |
| **Compositional*** **parameters** |  |  |  |
| Initial fluid fraction | *n*_fl,0_ [-] | 0.85 – 0.1*z*_H_ | (Wilson et al. 2005c) |
| Fixed charge density | *c*_FCD,0_ [mEq/ml] | -0.1*z*_H_^2^ + 0.24*z*_H_ + 0.035 | (Wilson et al. 2007) |
| Collagen density fraction | *ρ*_z_ [-] | 1.4h*z*_H_^2^ – 1.1h*z*_H_ + 0.59 | (Wilson et al. 2007) |
| **Material properties** |  |  |  |
| Nonfibrillar matrix modulus | $E_{\mathrm{nf}}$ [MPa] | 0.315 | (Wilson et al. 2005c) |
| Poisson ratio of the nonfibrillar matrix | $\nu_{\mathrm{nf}}$ [-] | 0.01 | (Wilson et al. 2005c) |
| Total number of fibrils (2 primary, 5 secondary) | *totf* | 9 | (Wilson et al. 2005c) |
| Fibril ratio between primary and secondary fibrils | *C* [-] | 3.009 | (Wilson et al. 2005c) |
| Fibril damping coefficient | *η* [MPa s] | 1418 | (Wilson et al. 2005a) |
| Strain-dependent fibril network modulus | $E_{\varepsilon}$ [MPa] | 867.7 | (Wilson et al. 2005c; Tanska et al. 2018) |
| Initial fibril network modulus | $E_{0}$ [MPa] | 2.737 | (Wilson et al. 2005a) |
| Initial permeability | *k*_0_ [mm^4^N^-1^s^-1^] | 1.522 | (Wilson et al. 2005c) |
| Strain-dependent permeability coefficient | *M* [-] | 1.339 | (Wilson et al. 2005c; Tanska et al. 2018) |
| External activity coefficient | $\gamma_{\mathrm{ext}}^{\pm}$ [-] | 0.757 | (Huyghe et al. 2003; Wilson et al. 2005c) |
| Material constant for chemical expansion | *a*_0_ [MPa ∙ ml/mEq] | 1.23 | (Wilson et al. 2005c) |
| Material constant for chemical expansion | *κ* [M^-1^] | 11.26 | (Wilson et al. 2005c) |
| Molar gas constant | R [J/mol K] | 8.3145 |  |
| Absolute temperature | T [K] | 293 | (Wilson et al. 2005c) |
| External salt concentration | $c_{\mathrm{ext}}$ [M] | 0.15 | (Wilson et al. 2005c) |
| External osmotic coefficient | $\phi_{\mathrm{ext}}$ [-] | 0.908 | (Huyghe et al. 2003; Wilson et al. 2005c) |

**z*_H_ is a variable for which *z_H_* = 0 at the impacted superficial zone and *z_H_* = 1 at the bottom of the sample.

Visualization of the initial fibril orientation, fixed charge density and converted proteoglycan concentration, water fraction and collagen fraction are shown in Fig. S1.


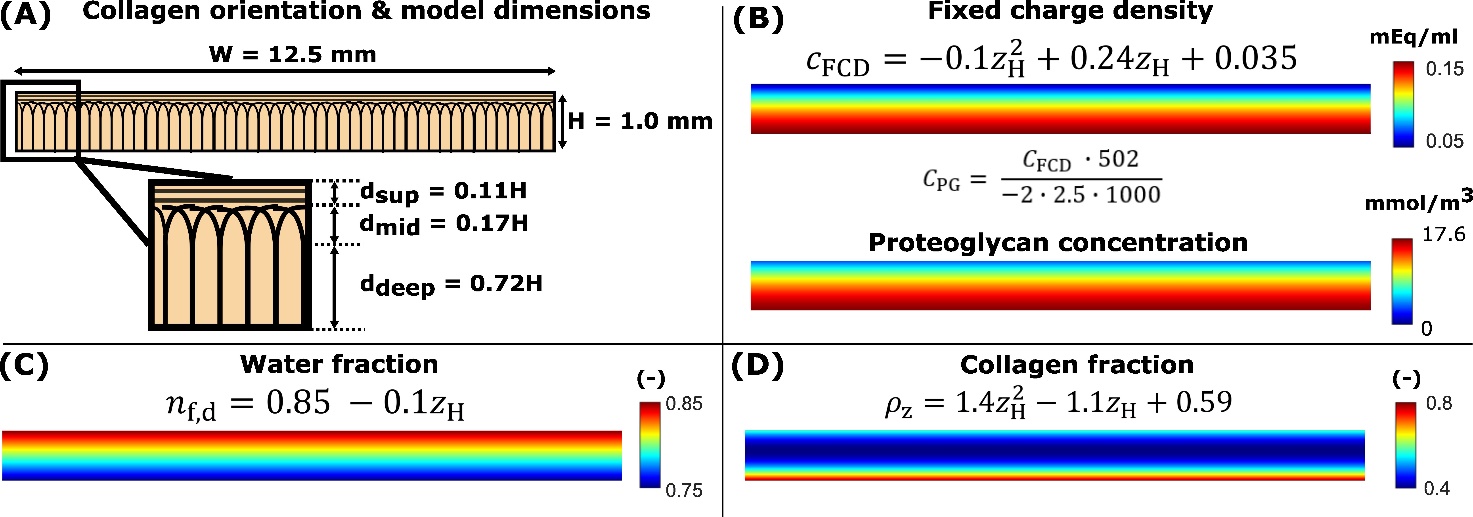


**Figure S1.** Initial **A)** collagen orientation (“Benninghoff type” arcade fibril architecture), **B)** fixed charge density and proteoglycan concentration calculated from based on the fixed charge density (Orozco et al. 2022), **C)** water fraction, and **D)** initial collagen density fraction in the mechanical impact model. In the figure, *z*_H_ is a variable defining depth-dependent distributions for which z_H_ = 0 at the impacted superficial zone and *z*_H_ = 1 at the bottom of the sample.

**S2 Sensitivity analysis for peak impact force and loading rate**

Sensitivity analysis for peak impact forces and loading rate was conducted to analyze changes in maximum shear strain distribution, pore pressure, and cell damage. Results of the sensitivity analysis are presented in Fig. S2 and Fig. S3.

Increasing peak impact force resulted in higher maximum shear strains mostly through cartilage depth (Fig S2 A-C). With the cell damage function as defined in Eq. 11, high impact force (6000N) caused the most drastic cell damage in the superficial zone as did also low impact force (2000N). Notably, with 6000N peak impact force more cell damage was observed in the deep zone compared to 4000N impact.


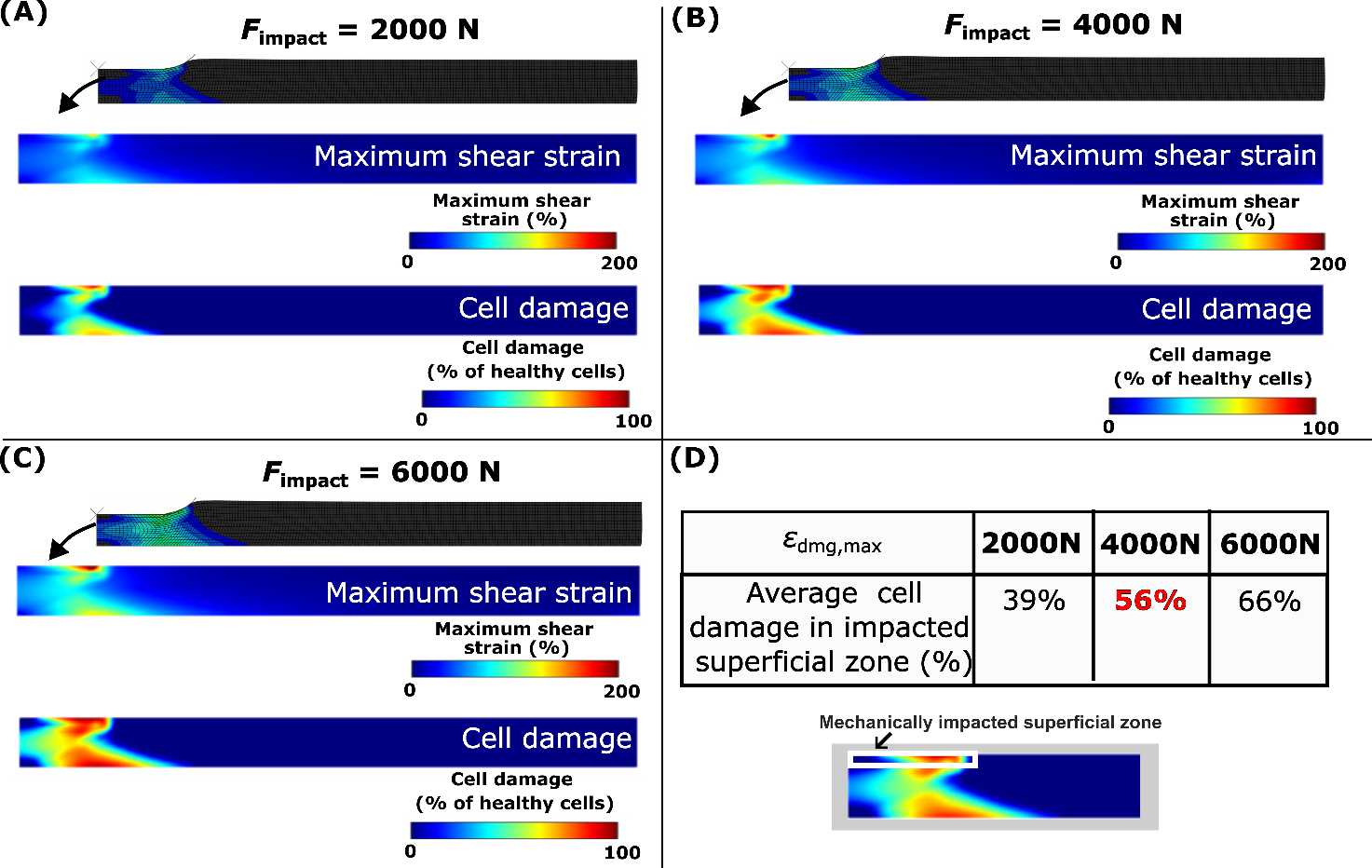


**Figure S2. Results of the sensitivity analysis for peak impact forces.** Maximum shear strain and cell damage distributions after impact with **A)** 2000N **B)** 4000N and **C)** 6000N peak impact forces. In the mechanically impacted superficial zone, **D)** average cell damage was increased as a function of peak impact force.


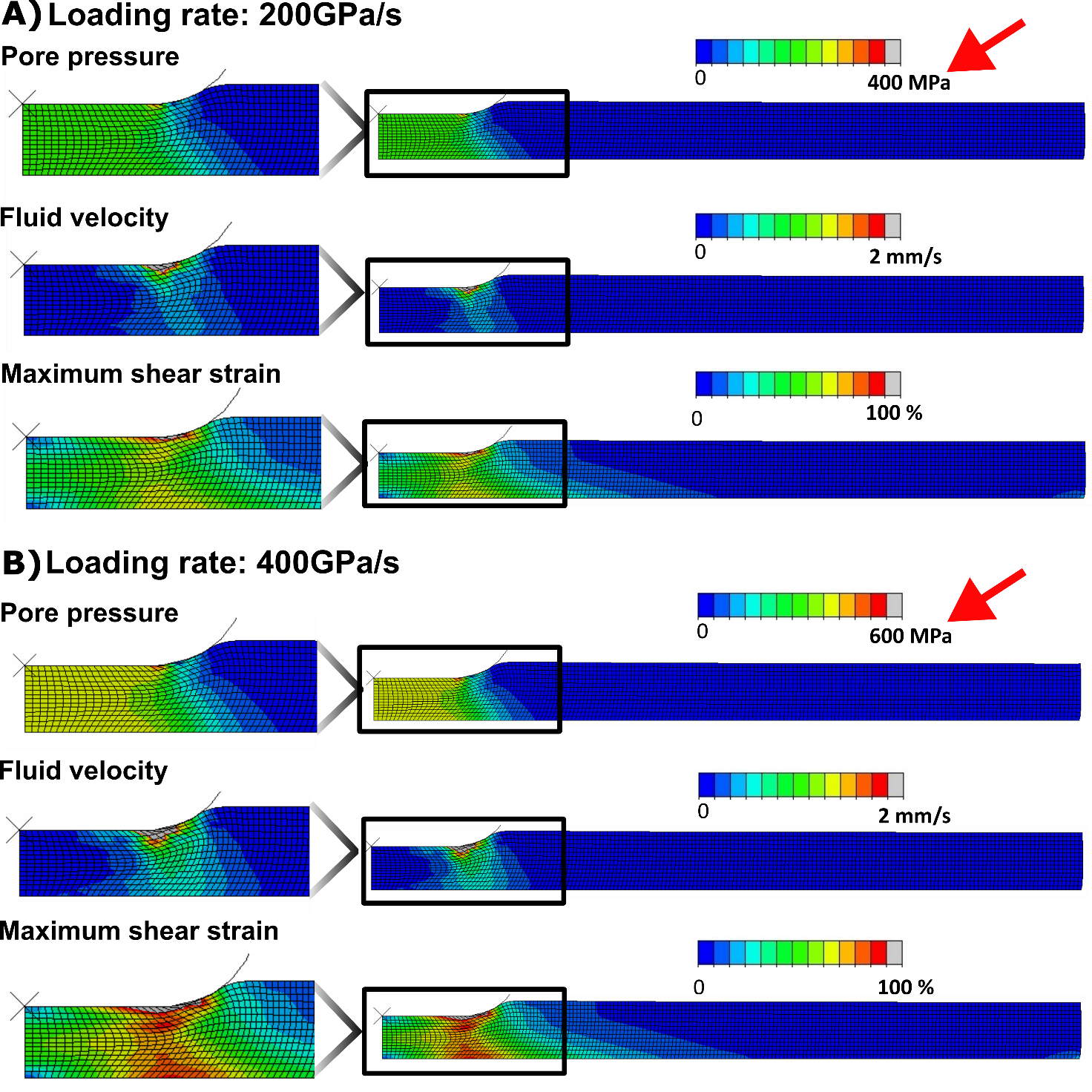


**Figure S3. Effect of loading rate to pore pressure, fluid velocity, and shear strain.** Pore pressure, fluid flow and maximum shear strain distributions after impact with **A)** 400GPa/s loading rate (4000N peak impact force in 0.5ms) **B)** 200GPa/s loading rate (2000N peak impact force in 0.5ms).

**S3 Green Lagrangian strain tensor and maximum shear strain**

Green-LaGrange tensor $\boldsymbol{E}$ is defined as

$\boldsymbol{E}\boldsymbol{=}\frac{1}{2}\left( \boldsymbol{C}\boldsymbol{-}\boldsymbol{I} \right)\boldsymbol{=}\frac{1}{2}\left( \boldsymbol{F}\boldsymbol{'}\boldsymbol{F}\boldsymbol{-}\boldsymbol{I} \right)$**,**

where $\boldsymbol{C}$ is the Cauchy-Green deformation tensor, $\boldsymbol{F}$ is the deformation gradient tensor, and $\boldsymbol{I}$ is the second order unit tensor. By calculating the eigenvalues of Green-Lagrange strain tensor $\varepsilon_{p,i}$, the maximum shear strain $\varepsilon_{\max}$ was determined as

$\varepsilon_{\max}=\max\left\{ |\varepsilon_{p,1} \right.-\varepsilon_{p,2}|,\left| \varepsilon_{p,1}-\varepsilon_{p,3} \right|,\left. |\varepsilon_{p,2}-\varepsilon_{p,3}| \right\}$.

**S4 Mesh convergence analysis**

Mesh convergence was verified by analyzing the maximum shear strains and cell damage distribution triggering cell death and proteoglycan degeneration. Based on the mesh convergence analysis, a mesh with 2506 elements was chosen for the rest of the model simulations. Meshes, maximum shear strains and average cell damage in the mechanically impacted superficial zone are presented in Fig. S3.


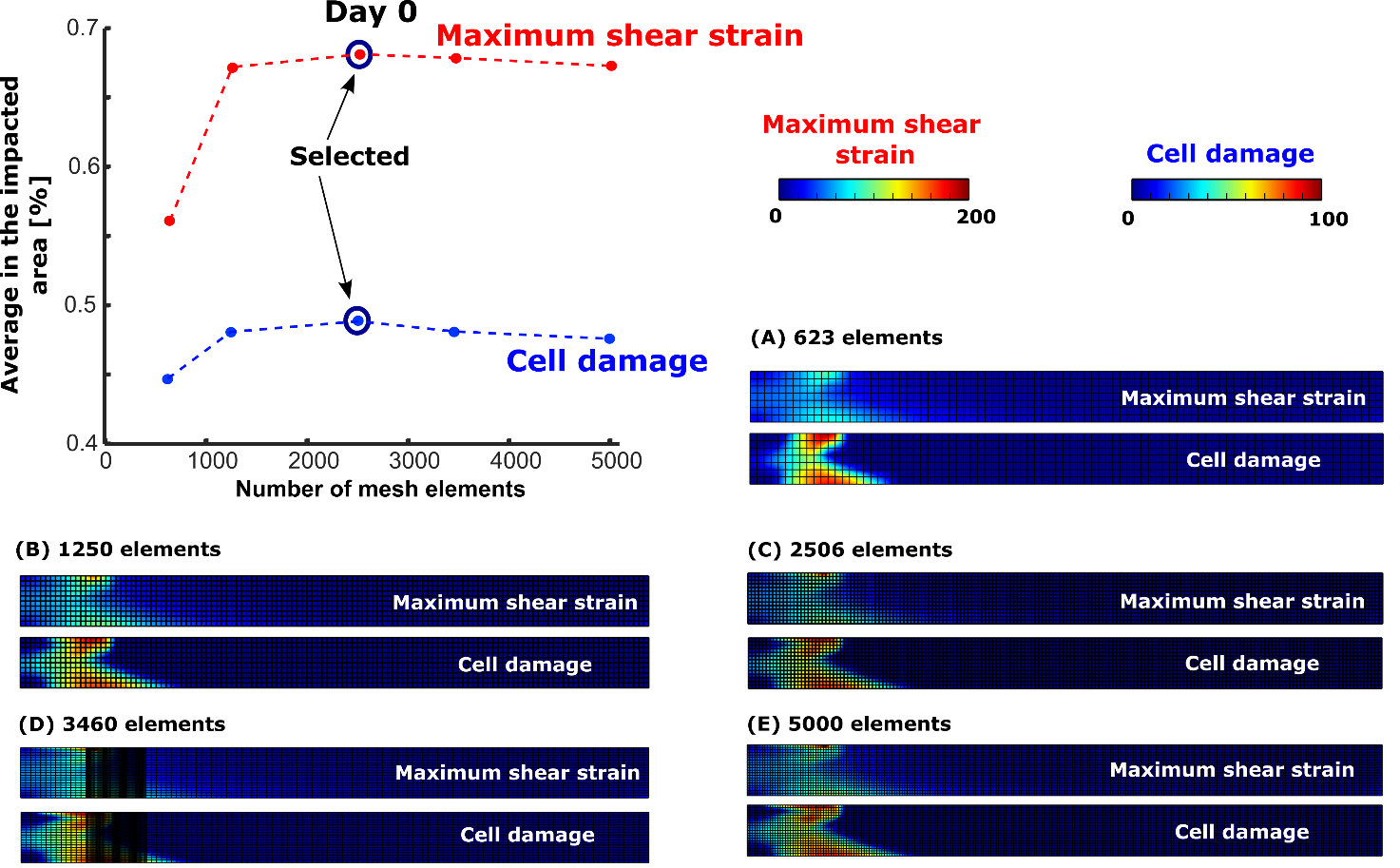


**Figure S4. Mesh convergence analysis.** Mesh convergence analysis was verified with 5 different meshes with **A)** 623, **B)** 1250, **C)** 2506, **D)** 3460 and **E)** 5000 elements. Mesh with 2506 elements was chosen for further analysis since only minor changes in maximum shear strains and cell damage were observed in the mechanically impacted superficial zone.

**S5 Effective diffusion, and proteoglycan degeneration in the biochemical model**

Proteoglycan degeneration was controlled via cell damage and increased proteolytic enzyme (aggrecanase) production accelerated by proteolytic enzyme net stimulus function *S* (Eq. 15). Proteolytic enzyme concentration $C_{\mathrm{aga}}$was modeled as:

|  | $\frac{\partial C_{\mathrm{aga}}}{\partial t}=D_{eff,aga}\nabla^{2}C_{\mathrm{aga}}+k_{4}S -k_{5}C_{\mathrm{aga}},$ | (S1) |
| --- | --- | --- |

where $D_{eff,aga}$ is the effective diffusivity of enzymes, $k_{4}$ is the stimulus rate coefficient for proteolytic enzyme production (Young et al. 2005; Kar et al. 2016a), $S$ is the stimulus term and $k_{5}$ is the aggrecanase proteolytic rate coefficient (Yamamoto et al. 2014; Kar et al. 2016a). For more detailed description of the model parameters, readers are referred to Kar et al. (Kar et al. 2016a).

Proteoglycan degeneration was modeled through the reaction term $R_{\mathrm{PG}}$ (synthesis/sink terms $R_{PG, syn}$ and $R_{PG, sink}$) as shown in Eq. 12:

|  | ${R_{\mathrm{PG}}=R}_{PG, syn}-R_{PG, sink}= {{(C}_{c,h}+C_{c,dmg})P}_{\mathrm{PG}}\left( 1-0.9z \right)\left( 1-\frac{C_{\mathrm{PG}}}{C_{\mathrm{target}}} \right) - {C_{\mathrm{aga}}k}_{3}\frac{C_{\mathrm{PG}}}{C_{\mathrm{PG}}+K_{m,aga}},$ | (S2) |
| --- | --- | --- |

where $C_{c,h}$ is the concentration healthy cells, $C_{c,dmg}$ is the concentration of damaged cells, $P_{\mathrm{PG}}$ is the basal aggrecan production rate (Kar et al. 2016a), $C_{\mathrm{PG}}$ is the proteoglycan concentration, $C_{\mathrm{aga}}$ is the aggrecanase concentration, $k_{3}$ is the catalytic rate constant for proteoglycans (Wittwer et al. 2007; Kar et al. 2016b), $K_{m,aga}$ is the Michaelis constant for proteolytic enzymes (aggrecanase) (Wittwer et al. 2007; Kar et al. 2016a) and $C_{\mathrm{target}}$ is the target concentration for proteoglycan in intact cartilage. In our modeling framework, the target aggrecan concentration was defined by scaling previously defined target concentration in Kar et al. (Kar et al. 2016b):

|  | $C_{\mathrm{target}}=\frac{C_{tar,K}}{C_{PG,max,K}}C_{PG,max},$ | (S3) |
| --- | --- | --- |

where $C_{tar,K}$ is the target proteoglycan concentration in Kar et al. (Kar et al. 2016b), $C_{PG,max,K}$ is the maximum proteoglycan concentration in Kar et al. (Kar et al. 2016b), and $C_{PG,max}$ is the maximum proteoglycan concentration calculated based on the fixed charge density (see Fig. S1B).

**S6 Sensitivity analysis for aggrecanase release from damaged cells**

Simulated proteoglycan loss was observed throughout the cartilage depth in the impacted area. Most of the proteoglycan loss was observed within 7 days after injury, and the lowest PG content was located at the impacted superficial zone (Fig. 4 A-D). Increasing proteolytic enzyme stimulus constant $k_{\mathrm{aga}}$ amplified the PG loss also in the deep regions of the cartilage experiencing acute cell damage. At day 14, the predicted PG content was 7, 14 and 21% lower in the impacted region when compared to intact region with $k_{\mathrm{aga}}=1.8 \cdot{10}^{-20}$ mol, $k_{\mathrm{aga}}=3.6 \cdot{10}^{-20}$ mol, and $k_{\mathrm{aga}}=5.4 \cdot{10}^{-20}$ mol, respectively (Fig. S3 E).


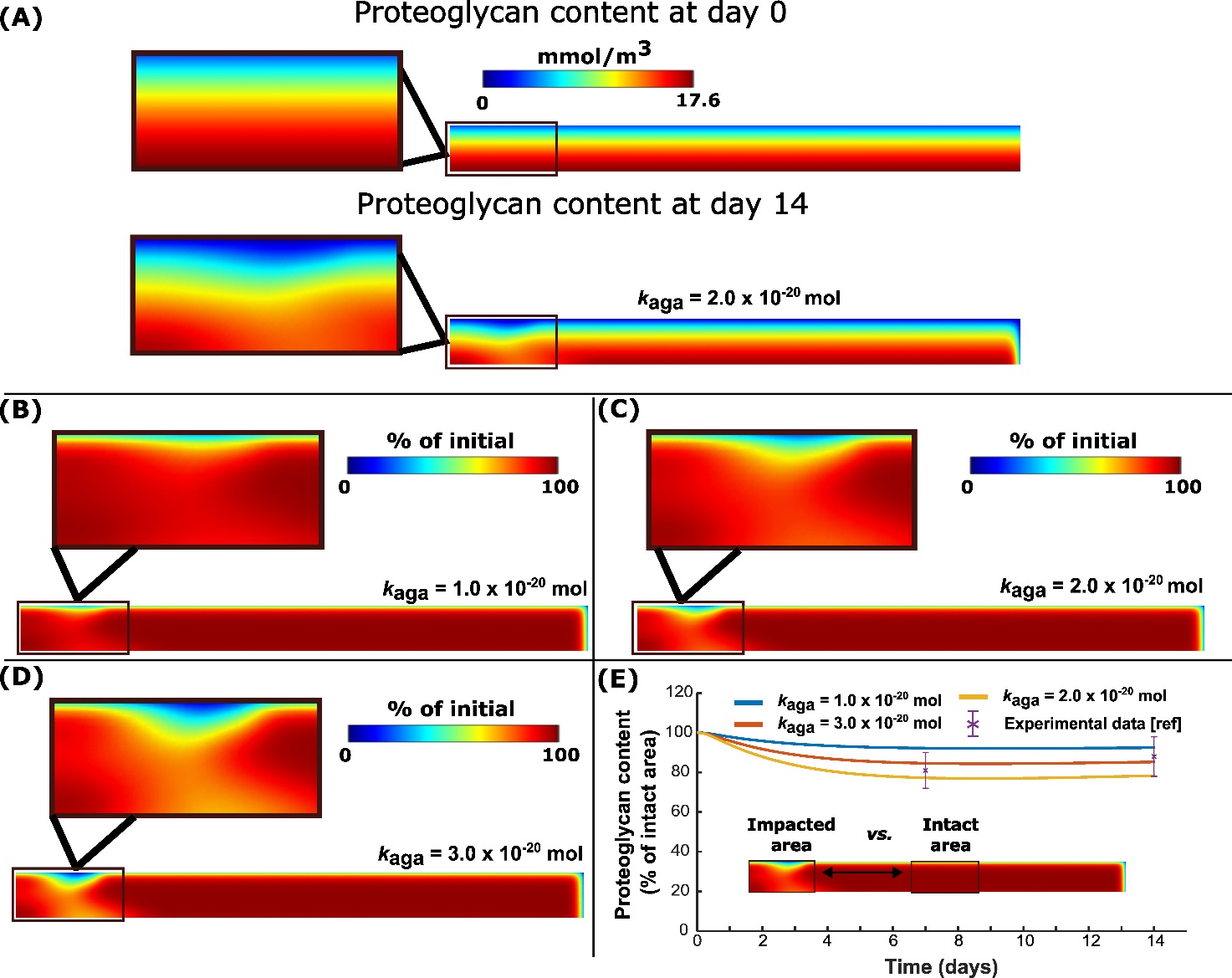


**Figure S5. Simulated cartilage proteoglycan degeneration without treatment.** **A)** Proteoglycan concentration distributions and **B-D)** relative proteoglycan content at day 14 when proteolytic enzyme release from damaged cells was selected as **A)** $k_{\mathrm{aga}}=1.8 \cdot{10}^{-20}$ mol, when **B)** $k_{\mathrm{aga}}=3.6 \cdot{10}^{-20}$ mol, and when **C)** $k_{\mathrm{aga}}=5.4 \cdot{10}^{-20}$ mol. **D)** relative proteoglycan content between impacted and intact area. With $k_{\mathrm{aga}}=3.6 \cdot{10}^{-20}$ mol our model was capable to replicate relative proteoglycan content as observed in the experiments.

**References**

Huyghe JM, Houben GB, Drost MR, van Donkelaar CC (2003) An ionised/non-ionised dual porosity model of intervertebral disc tissue. Biomech Model Mechanobiol 2:3–19. https://doi.org/10.1007/s10237-002-0023-y

Julkunen P, Kiviranta P, Wilson W, et al (2007) Characterization of articular cartilage by combining microscopic analysis with a fibril-reinforced finite-element model. J Biomech 40:1862–1870. https://doi.org/10.1016/j.jbiomech.2006.07.026

Kar S, Smith DW, Gardiner BS, et al (2016a) Modeling IL-1 induced degradation of articular cartilage. Arch Biochem Biophys 594:37–53. https://doi.org/10.1016/j.abb.2016.02.008

Kar S, Smith DW, Gardiner BS, et al (2016b) Modeling IL-1 induced degradation of articular cartilage. Arch Biochem Biophys 594:37–53. https://doi.org/10.1016/j.abb.2016.02.008

Martin JA, McCabe D, Walter M, et al (2009) N-acetylcysteine inhibits post-impact chondrocyte death in osteochondral explants. Journal of Bone and Joint Surgery - Series A 91:1890–1897. https://doi.org/10.2106/JBJS.H.00545

Orozco GA, Eskelinen ASA, Kosonen JP, et al (2022) Shear strain and inflammation-induced fixed charge density loss in the knee joint cartilage following ACL injury and reconstruction: A computational study. Journal of Orthopaedic Research 40:1505.1522. https://doi.org/10.1002/jor.25177

Tanska P, Julkunen P, Korhonen RK (2018) A computational algorithm to simulate disorganization of collagen network in injured articular cartilage. Biomech Model Mechanobiol 17:689–699. https://doi.org/10.1007/s10237-017-0986-3

Wilson W, Huyghe JM, Van Donkelaar CC (2007) Depth-dependent compressive equilibrium properties of articular cartilage explained by its composition. Biomech Model Mechanobiol 6:43–53. https://doi.org/10.1007/s10237-006-0044-z

Wilson W, Van Donkelaar CC, Van Rietbergen B, et al (2004) Stresses in the local collagen network of articular cartilage: A poroviscoelastic fibril-reinforced finite element study. J Biomech 37:357–366. https://doi.org/10.1016/S0021-9290(03)00267-7

Wilson W, Van Donkelaar CC, Van Rietbergen B, et al (2005a) Erratum: A fibril-reinforced poroviscoelastic swelling model for articular cartilage (Journal of Biomechanics (2005) 38 (1195-1204) PII: S0021929004003367 and DOI: 10.1016/S0021-9290(03)00267-7). J Biomech 38:2138–2140. https://doi.org/10.1016/j.jbiomech.2005.04.024

Wilson W, Van Donkelaar CC, Van Rietbergen B, Huiskes R (2005b) A fibril-reinforced poroviscoelastic swelling model for articular cartilage. J Biomech 38:1195–1204. https://doi.org/10.1016/j.jbiomech.2004.07.003

Wilson W, Van Donkelaar CC, Van Rietbergen B, Huiskes R (2005c) A fibril-reinforced poroviscoelastic swelling model for articular cartilage. J Biomech 38:1195–1204. https://doi.org/10.1016/j.jbiomech.2004.07.003

Wittwer AJ, Hills RL, Keith RH, et al (2007) Substrate-dependent inhibition kinetics of an active site-directed inhibitor of ADAMTS-4 (aggrecanase 1). Biochemistry 46:6393–6401. https://doi.org/10.1021/bi7000642

Yamamoto K, Owen K, Parker AE, et al (2014) Low density lipoprotein receptor-related protein 1 (LRP1)-mediated endocytic clearance of a disintegrin and metalloproteinase with thrombospondin motifs-4 (ADAMTS-4): Functional differences of non-catalytic domains of ADAMTS-4 and ADAMTS-5 in LRP1 binding. Journal of Biological Chemistry 289:6462–6474. https://doi.org/10.1074/jbc.M113.545376

Young DA, Lakey RL, Pennington CJ, et al (2005) Histone deacetylase inhibitors modulate metalloproteinase gene expression in chondrocytes and block cartilage resorption. Arthritis Res Ther 7:. https://doi.org/10.1186/ar1702
